# Is Laser Therapy an Adjuvant in the Treatment of Peri-Implant Mucositis? A Randomized Clinical Trial

**DOI:** 10.3390/diagnostics13061192

**Published:** 2023-03-21

**Authors:** Luminița Lazăr, Timea Dakó, Izabella-Éva Mureșan, Mircea Suciu, George-Alexandru Maftei, Monica Tatarciuc, Ana-Petra Lazăr

**Affiliations:** 1 Department of Periodontology, George Emil Palade University of Medicine, Pharmacy, Science, and Technology of Targu Mures, 540139 Targu Mures, Romania; 2 Department of Odontology and Oral Pathology, George Emil Palade University of Medicine, Pharmacy, Science, and Technology of Targu Mures, 540139 Targu Mures, Romania; 3 County Emergency Clinical Hospital of Targu Mures, 540139 Targu Mures, Romania; 4 Department of Oral Rehabilitation and Occlusology, George Emil Palade University of Medicine, Pharmacy, Science, and Technology of Targu Mures, 540139 Targu Mures, Romania; 5 Department of Dento-Alveolar Surgery and Oral Pathology, Grigore T. Popa University of Medicine and Pharmacy Iasi, 700115 Iasi, Romania; 6 Department of Oral Implantology, Removable Dentures and Technology, Grigore T. Popa University of Medicine and Pharmacy Iasi, 700115 Iasi, Romania

**Keywords:** laser, peri-implant mucositis, peri-implantitis, adjuvant therapy

## Abstract

(1) Background: Early diagnosis and treatment of peri-implant mucositis may reduce inflammatory markers and halt the progression of the condition to peri-implantitis. Adjunctive laser treatment may have therapeutic benefits that are not yet well known. The aim of this study was to determine the advantages and limitations of laser therapy as an adjuvant in the treatment of peri-implant mucositis. (2) Methods: A total of 42 patients with at least 2 implants situated in different hemiarches were included in this study and divided into two groups: G1 (received laser therapy) and G2 (no laser therapy). Periodontal health status indices were recorded at the initial moment (T0), and all patients underwent non-surgical debridement therapy accompanied by oral hygiene training. In patients from group G1, one implant site received adjuvant laser therapy (subgroup IL), and the other one did not receive active laser light (IC). The plaque index (PI), probing pocket depth (PPD), and bleeding on probing (BOP) values recorded after 3 months (T1) and 6 months (T2) were analyzed and compared with those at T0. (3) Results: PI values considerably reduced at moment T1 and T2 for both G1 and G2 (*p* = 0.0031). PPD was also reduced, but the difference between the groups and the three recording moments was not statistically significant. Statistically significant differences were found when comparing the BOP values between G1 IL and G1 IC for T0/T1 (*p* = 0.0182) and T1/T2 (*p* < 0.0001), but there was no significant difference between G2 and G1 IL or G1 IC. (4) Conclusions: Laser therapy as an adjunct to conventional treatment of peri-implant mucositis leads to a statistically significant reduction in bleeding on probing at 3-month and 6-month re-evaluations. Moreover, it leads to an evident reduction in probing depth but with no statistical significance. These results should be interpreted with caution, and more in-depth research should be performed to create a complete laser therapy protocol for peri-implant mucositis.

## 1. Introduction

Implant-assisted edentulous therapy has become a routine treatment in dentistry nowadays as it is increasingly widespread among clinicians worldwide [1]. Although dental implants are a reliable treatment method with a high survival rate (94.6%), the prevalence of peri-implant disease has been reported by several longitudinal and cross-sectional studies [2,3,4,5,6,7,8,9]. Peri-implant mucositis has a prevalence of 29.48% at implant level and 46.83% at patient level, and for peri-implantitis, the prevalences are 9.25% and 19.83%, respectively [10].

Over the years, several studies have defined implant success criteria [11,12,13]. The success of dental implants is determined through a comprehensive assessment, reported at different levels: implant, peri-implant soft tissues, prosthetic work, and patient. Judging by the state of the implant, success criteria are absence of mobility, pain, radiolucency, and peri-implant bone loss (more than 2 mm in the first year). For peri-implant soft tissues, the success criteria should be the absence of suppuration and bleeding. Implant success is reached when the prosthetic work has no technical/prosthetic complications and has provided adequate functional and esthetic rehabilitation. For the patient, the success criteria are the satisfaction provided by the esthetics and the ability to perform the masticatory function without any discomfort and/or paresthesia [13].

Most of the complications associated with dental implants are inflammatory conditions of the soft and hard tissues around them, which are induced by the accumulation of bacterial biofilm [14]. Such conditions, which have been called peri-implant mucositis and peri-implantitis, must be clearly defined and differentiated from the peri-implant health status, to establish a proper diagnosis and institute an appropriate treatment.

The new classification of periodontal and peri-implant diseases elaborated and published by American and European researchers is intended to simplify and clarify their diagnosis [15,16]. An element of novelty was the inclusion of peri-implant conditions within this classification, starting from the idea that the periodontologist is the clinician to diagnose and treat them. This classification provides specific criteria to accurately define peri-implant status in daily practice: signs of gingival inflammation, bleeding on probing (BOP), probing pocket depth compared to previous visits (PPD), and radiographically detectable bone loss (RBL) [17].

Peri-implant health is defined by the absence of signs of peri-implant soft tissue inflammation, the absence of bleeding and/or suppuration on gentle probing, the absence of increased probing depth (PPD) compared to previous visits, and the absence of radiographic bone loss (RBL) beyond the changes of the crestal bone level that appeared due to initial bone remodeling after implant placement [18].

Peri-implant mucositis is characterized by the presence of bleeding and/or suppuration on gentle probing with or without increased probing depth compared with previous examinations and the absence of additional changes in radiographic bone loss that occurred after initial bone remodeling [19].

The means by which peri-implantitis diagnosis is made depends on the presence or absence of previous records. Using previous records, peri-implantitis is defined by the presence of signs of bleeding and/or suppuration on mild probing, increased PPD compared with previous examinations, and the presence of RBL versus crestal bone level changes after initial bone remodeling that should not be higher than 2 mm. In the absence of previous radiographic records, the signs used to define a case of peri-implantitis are the presence of bleeding and/or suppuration on gentle probing, and PPD ≥ 6 mm and RBL ≥ 3 mm apical to the most coronal part of the intraosseous portion of the implant [20].

The pathological process always begins with peri-implant mucositis, which affects only the soft tissue around the implant. This pathological condition is reversible when detected early and treated properly [21,22,23]. The standard protocol in the treatment of peri-implant mucositis consists of training and monitoring the patient regarding oral hygiene measures and instituting non-surgical therapy [24,25,26]. For the non-surgical treatment of peri-implant mucositis, different methods have been studied such as techniques that improve dental plaque removal, locally applied antiseptics, generally administered antibiotics, probiotics, or the use of mouthwashes containing chlorhexidine [27,28,29,30,31,32,33].

Regarding the use of laser therapy as an adjuvant in the treatment of peri-implant mucositis, the results of the studies conducted are controversial [34,35,36,37]. That is the main reason why we aimed to evaluate the advantages and limitations of using laser therapy in the treatment of peri-implant mucositis in this study.

## 2. Materials and Methods

### 2.1. Study Design

This clinical study was conducted as a double-blind, randomized clinical trial.

### 2.2. Selection of Patients

**Figure 1:**
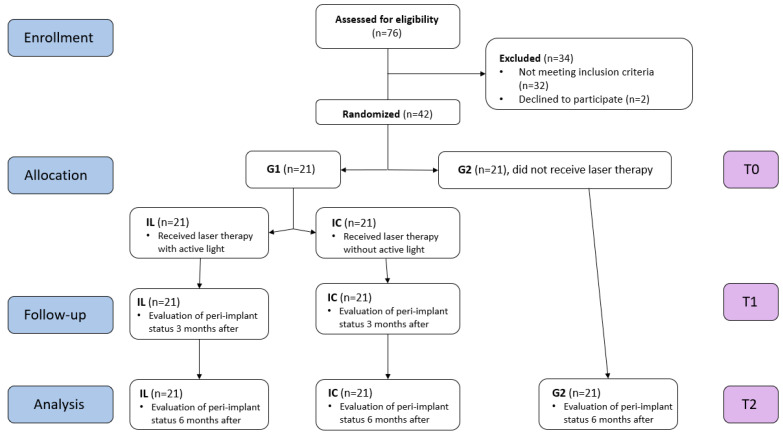
Flow diagram of the study.

Out of 76 adult patients with dental implant restorations who presented at the dental office in Targu Mures (Romania) for periodic check-ups, between 3 January 2021 and 22 December 2022, we selected 42 patients who met the following inclusion criteria:

- Presence of at least one implant on two different hemiarches;
- Implants must be pillars of fixed prosthetic works;
- Presence of bacterial plaque and signs of inflammation of the peri-implant gingival tissue.
- The exclusion criteria were the following:
- Presence of radiographically detectable bone loss after the initial remodeling of the bone;
- Presence of systemic diseases with an impact on the periodontal tissues (diabetes, immunological diseases, acute articular rheumatism, tuberculosis, etc.);
- Pregnancy or breastfeeding;
- Non-surgical peri-implant treatment performed in the last 6 months;
- Antibiotic treatment in the last 6 months;

The use of non-steroidal anti-inflammatory drugs (Figure 1).

The patients were informed about the procedure and about the fact that they could leave this study at any time, and signed an informed consent. Sample size was determined using the power analysis calculation. A total of 17 patients per group were estimated to provide 90% power for the detection of 1.0 mm of difference in the probing pocket depth (PPD) between the two groups with a standard deviation of 0.8 mm, 0.05 type I error, and 0.1 type II error. Considering the potential withdrawal of patients, we sought to enroll at least 21 patients per group.

### 2.3. The Periodontal Protocol

The periodontal status was evaluated by a periodontologist other than the one who performed the laser therapy.

Patients who, at the check-ups, after the completion of the fixed prosthetic treatment with implant support, showed accumulation of bacterial plaque and signs of peri-implant gingival inflammation underwent a new periodontal examination. After the radiographic examination proved the absence of bone loss and after the initial physiological remodeling of the bone, the following indices were recorded in a periodontal record:

- Plaque index (PI): the presence (+) or absence (−) of bacterial plaque on the buccal, lingual, mesial, and distal surfaces following the application of a plaque disclosing solution. The PI value was calculated by dividing the sum of all surfaces presenting dental plaque by the total number of surfaces examined, multiplied by one hundred;
- Probing pocket depth (PPD): the distance from the gingival margin to the apical limit of the peri-implant gingival groove measured in 6 places (mesio-buccal/centro-buccal/disto-buccal/mesio-oral/centro-oral/disto-oral) with a constant force;
- Bleeding on probing (BOP): by giving the following scores: 1, minimal punctate bleeding; 2, linear bleeding or in drops; 3, spontaneous or profuse bleeding, with or without suppuration [38].

Patients were divided into two groups:

- Group 1: 21 patients who received instructions regarding dental plaque removal and underwent scaling around the implant surface using titanium curettes. Only one out of the two implants each patient had benefited from laser treatment. The peri-implant status was evaluated at the time of the initial examination (T0), three months after (T1), and 6 months after (T2).
- Group 2: 21 patients who received instructions regarding dental plaque removal and underwent scaling around the implant surface using titanium curettes. The peri-implant status was evaluated at the time of the initial examination (T0) and at 6 months (T2).

### 2.4. The Laser Protocol

Laser therapy was randomly performed for one of the implants (IL) for each patient in group 1. For the second implant, located on another hemiarch, the same protocol was followed but without active light (IC). The patients and the periodontologist who evaluated the periodontal status were informed that only one of the implants benefited from laser therapy without specifying which one. During the irradiation, both the patient and the doctor wore protective glasses. The peri-implant sites were irradiated at moments T0 and T1 by the same clinician for the same implant site.

Laser therapy was performed with a dental diode laser (Prime, Litemedics, Lambda SpA, Milano, Italy), with a power of 12 Watt, in pulsed system and operating wave of 980 nm, using the working mode “periodontology”. A 320-micrometer optical fiber was inserted in the gingival sulcus and moved in a mesio-distal direction, both on the buccal surface and on the lingual surface, for 30 s.

### 2.5. Statistical Analysis

All data were collected in Microsoft Excel worksheets (Microsoft Corporation, Washington, DC, USA, 2018). Statistical analysis was performed with GraphPad Prism version 8.0.0 for Windows (GraphPad Software, San Diego, CA, USA). For each group of data, descriptive statistics such as mean, standard deviation, median, minimum, and maximum value were determined. Data normality was determined by the Kolmogorov–Smirnov test. The difference between the values of the clinical indices recorded at T0, T1, and T2 was determined using Fischer’s and ANOVA tests. The significance level chosen was set at 0.05.

## 3. Results

Patients selected to participate in this study, based on the inclusion and exclusion criteria, were aged between 27 and 58 years. Group 1 consisted of 12 women with mean age of 43 years and 9 men with a mean age of 45 years. Group 2 included 11 women with an average age of 46 years and 10 men with an average age of 42 years. For each patient, the values of the main indicators of peri-implant health status were recorded: plaque index (PI), bleeding on probing (BOP), and probing pocket depth (PPD). The mean values recorded for PI, BOP, and PPD for each group are presented in Table 1.

**Figure 2:**
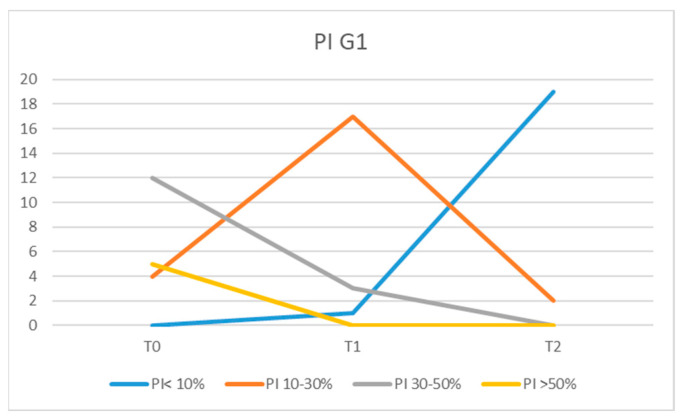
Values of PI in G1 at T0, T1, and T2.

At the initial examination (moment T0), high PI values were recorded for most patients, with an average of 41.07% in the G1 group and 42.85% in the G2 group. In the G1 group at moment T0, 5 patients had PI values >50%, 12 recorded PI values of 30–50%, and 4 patients had PI between 10 and 30%. After a rigorous prophylactic cleaning session and patient instruction regarding dental plaque control, the PI values recorded at moments T1 and T2 were lower for all patients in the G1 group. Thus, at moment T1, no patient had a PI > 50%, and 3 had a PI value of 30–50%; for 17 patients, we recorded PI values of 10–30%, and 1 patient had a PI < 10% (Figure 2).

**Figure 3:**
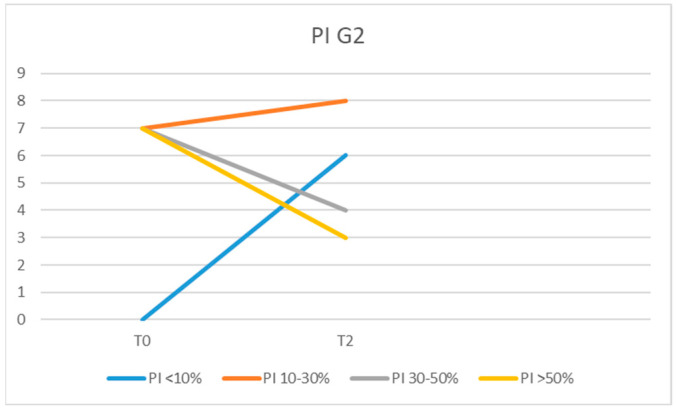
PI values in G2 at moments T0 and T2.

The values recorded for PI in patients from group G2 at T2 moment, were low in most patients compared with the T0 moment. The values recorded at T2 were PI > 50% for three patients, PI = 30–50% for four, PI = 10–30% in eight of the patients, and PI < 10% in six of them (Figure 3). However, three patients had PI values similar to those recorded at moment T0, and two patients showed higher PI values.

When comparing the average values of the plaque index between G1 and G2 at moment T2, a statistically significant reduction (*p* = 0.0311) was observed in patients from group G1.

**Figure 4:**
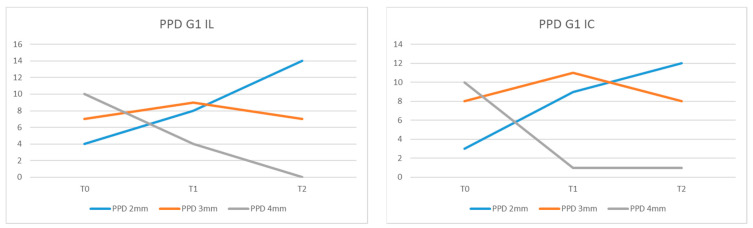
PPD values in G1 IL and G1 IC at moments T0, T1, and T2.

The values recorded for PPD in the peri-implant sites in group G1 IL at moment T0 were 4 mm for 10 patients, 3 mm for 7 patients, and 2 mm for 4 of them. At moment T1, we recorded PPD = 4 mm in four patients, PPD = 3 mm in nine, and PPD = 2 mm in eight of the examined patients. At the 6-month examination (T2), the patients presented the following values: 1 had PPD = 4 mm, 8 had PPD = 3 mm, and 12 of them had PPD = 2 mm (Figure 4).

**Figure 5:**
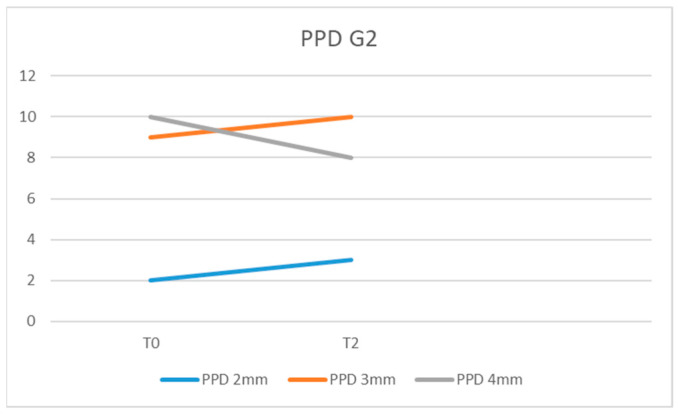
PPD values in G2 at moments T0 and T2.

In G2, we recorded at the initial examination (T0) PPD = 4 mm in 10 of the examined patients, PPD = 3 mm in 9, and PPD = 2 mm in 2 of them (Figure 5).

The mean probing pocket depth (PPD) in the G1 IL group was 3.28 mm at T0, 2.80 mm at T1, and 2.33 mm at T2. For G1 IC, the values recorded for PPD were 3.33 mm (T0), 2.85 mm (T1), and 2.61 mm (T2). For G2, the average PPD values were 3.38 mm at T0 time and 3.23 mm at T2 time.

The difference between the mean values of PPD at T0, T1, and T2 between the G1 IL and IC groups was statistically insignificant (*p* = 0.48) as well as between G1 IL and G2 at the time of T2 (*p* = 0.4003). Comparing the mean values of PPD between G1 IL and G1 IC at T1 versus T0, no statistically significant difference was found (*p* = 0.48). Even when comparing the mean values of PPD for G1 IL and G1 IC, there was no statistically significant difference recorded at T2 compared with T1 (*p* = 0.194).

When recording the bleeding on probing (BOP), we found that all patients included in our study presented a higher score than one at moment T0.

**Figure 6:**
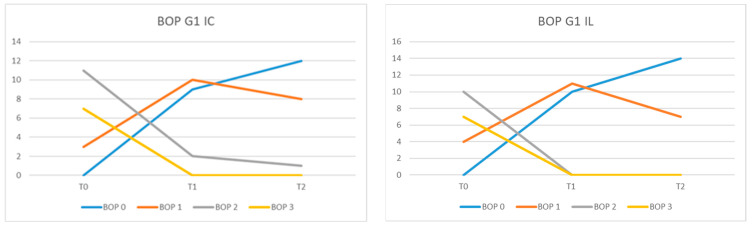
BOP values in group G1 Il and G1 IC at moments T0, T1, and T2.

In G1 IL, at moment T0, no patient had BOP = 0, 4 had BOP equal to 1, 10 had BOP = 2, and 7 had BOP = 3. At moment T1, 10 patients had BOP = 0 and 11 patients BOP = 1, and at time T2, 14 of them had BOP = 0, and 7 patients BOP = 1 (Figure 6).

**Figure 7:**
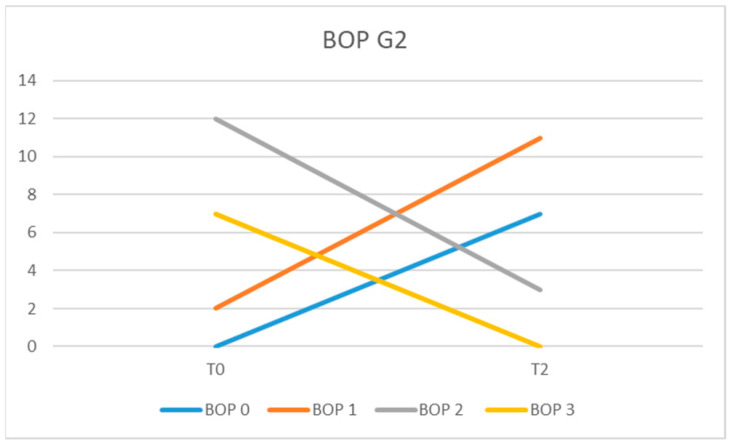
BOP values in group G at moments T0 and T2.

In the G2 group at the initial examination (T0), 7 patients presented BOP = 3; 12 had BOP = 2; and, in 2 patients, we recorded BOP = 1. At the 6-month examination (T2), the recorded scores for BOP were 2 for 3 patients, 1 for 11 patients, and 0 for 7 of them (Figure 7).

The average values of BOP at time T0 were 2.14 for the patients of the G1 IL group, 2.19 for those in the G1 IC group, and 2.23 for patients from group G2. In the G1 IL group, the mean BOP values were 0.52 (T1) and 0.33 (T2), while in the G1 IC they were 0.66 (T1) and 0.47 (T2). In the G2 group at time T2, the mean BOP value was 0.80.

The difference between the mean BOP values at moments T0, T1, and T2 between the G1 IL and IC groups was statistically significant (*p* = 0.0162). When comparing mean BOP values in G1 IL versus G1 IC, a statistically significant reduction was observed (*p* = 0.0182) in T1 versus T0 and a highly significant difference between T2 and T1 (*p* < 0.0001). Comparing the mean BOP values between G2 and G1 IL, the difference was not statistically significant (*p* = 0.0743) nor between G2 and G1 IC (0.0584).

## 4. Discussion

Implant treatments are becoming more and more frequent and so are the potential negative effects that can come with implant-associated pathologies such as peri-implant mucositis and peri-implantitis. A recent review examined the potential risk factors for implant failure and treatments available and stated that pocket depth reduction can be achieved in the short-term with laser, and air powder abrasive could aid in cleaning a contaminated implant surface. The authors also stated that plaque control, surgical pocket elimination, and bone recontouring are other efficient treatments for peri-implantitis [21].

In our study, we tried to create patient groups that were as homogeneous as possible in terms of age and sex, so that these demographics did not influence the study results.

To establish the diagnosis of peri-implant mucositis, we examined the patients clinically (PI, PPD, and BOP) and radiographically. In G1, these assessments were made for two implants located at a certain distance from each other to ensure an objective assessment of the peri-implant status. If the patient presented several implants, the two implants that had the highest values of the recorded periodontal indices were included. In G2, the implants that recorded the most advanced signs of peri-implant mucositis were included. In group 1, laser therapy was randomly applied to one implant (IL), so that we could compare the values of the periodontal indices for IL with those obtained from the implant that did not receive active light (IC) in the same patient. Thus, the evaluation of the laser therapy was an objective one without oral hygiene habits, which differ from one patient to another, influencing the results.

We used PI, which assesses the plaque accumulation in the entire oral cavity as a percentage, to have an overview of the oral hygiene of each patient. The results of our study showed that there were patients who were not monitored at three months (T1) and had PI values comparable or even higher than those at the time of T0 during the 6-month examination (T2). The findings that the mean values of PI for G2 at the time of T2 were not significantly lower than at T0 and that, at G1, the differences between the PI values at these times were significant prove the importance of repeated controls at intervals of 3 months in patients with implant therapy. The frequency of intervals between training sessions and professional cleaning usually varies between 3 and 6 months, and their frequency should be based on the risk profile of each patient [39]. Monk et al. observed that patients with a history of periodontal disease are more compliant regarding oral hygiene measures and with periodic check-ups [40]. Supportive therapy provides the clinician with the opportunity to monitor peri-implant status, and professional dental care improves peri-implant health and, hence, the success rate of dental implants. The patient’s informed consent form should include the accordance of the patient to comply with personal and professional peri-implant supportive therapy. Rokn et al. observed that after 5 years of implant loading without following a regular maintenance schedule, one in five patients presents with peri-implantitis [41].

In this study we recorded no statistically significant change in PPD recording between G1IL, G1IC, and G2 at moment T2. However, we recorded reductions in PPD values for most patients, which explain the remission of inflammatory phenomena. Al Rifaiy et al. observed a statistically significant decrease (*p* < 0.001) in PPD in patients who benefited from laser therapy, both when comparing the values obtained at 12 weeks with the initial one and when comparing those recorded in patients who did not benefit from it [42]. The same results of statistically significant reduction of PPD after using laser therapy were obtained by Lerario et al. [43]. The finding that 89% of the implant sites presented at initial levels of PPD higher than 4 mm may explain the difference to the results of our study, in which 71% of patients presented at an initial PPD = 4 mm and no value was higher than 4 mm.

For the BOP evaluation, we chose to use the variant of giving a score, proposed by American researchers [38], because it allows the quantification of bleeding on probing at each individual implant site. The percentage evaluation in the entire oral cavity would not have allowed us to evaluate the results of the laser therapy at each implant’s level. In our study, the finding that mean BOP values in G1 IL were significantly reduced at T2 compared with T0 and compared with G1 IC demonstrates that laser therapy can be an adjuvant in the treatment of peri-implant mucositis. Similar results were obtained by Al Rifaiy et al., who concluded that antimicrobial laser therapy, as an adjuvant in the treatment of peri-implant mucositis, is more effective than simple mechanical instrumentation [42]. In a study conducted on 125 implants, the authors observed significantly reduced values of PPD and BOP, with values ≤ 5%, in patients treated with laser [43]. Repeated adjunctive application of laser therapy at 0, 7, and 14 days at peri-implant sites produced significant clinical improvements after an observation period of at least 2 years [44]. The results of the study by Sánchez-Martos et al. showed that patients who received laser therapy as an adjunct to conventional treatment of mucositis had less bleeding at the 3-month reassessment than patients who received only conventional therapy (*p* < 0.001) [45].

Starting from the observation that in patients who received adjuvant laser therapy, BOP was positive at 44 sites at T0 and t 6 sites at 3 months (T1), while for patients who received only mechanical treatment, BOP was positive at 52 sites at T0 and t 28 of places at 3 months (T1), Tenore et al. considered that laser therapy can be used as an adjunct to mechanical therapy method [37]. Mariani et al. concluded that the additional use of laser showed small additional benefits in the treatment of peri-implant mucositis after a one-year observation period, which was not statistically significant [35].

On the other hand, the results of other clinical studies led the authors to the conclusion that the additional use of laser had no further positive influence on peri-implant healing compared with mechanical instrumentation as monotherapy [34,46]. Atieh et al. concluded that in the management of peri-implant mucositis, the combined use of diode laser and mechanical debridement provided no additional clinical advantage over mechanical debridement alone [47].

Adjunctive therapy such as laser or photodisinfection treatment could provide an auxiliary advantage in peri-implantitis, as was illustrated for periodontitis, especially in patients with other systemic pathologies, such as diabetes, myocardial infarction, or rheumatoid arthritis [48,49,50,51].

Early diagnosis of peri-implant mucositis and the application of effective therapeutic methods are preventive measures in the occurrence of peri-implantitis [52].

The limitations of our study consist of the small group of patients evaluated and the evaluation of only clinical indexes; microbiological or biochemical data could have offered a more complete image of laser treatment efficacy. Another limitation is lack of comparison with other adjuvant methods in order to assess treatment superiority.

Given that the data regarding adjuvant laser treatment of peri-implant mucositis are sparse and controversial, future clinical trials are needed to evaluate the potential benefit of this approach.

## 5. Conclusions

The peri-implant health status is directly correlated with the maintenance of oral hygiene; therefore, the clinician must give importance to supportive therapy in order to increase the success rate of dental implants.

Laser therapy as an adjunct to conventional treatment of peri-implant mucositis led to a statistically significant reduction in probing bleeding at 3-month and 6-month re-evaluations. When PPD ≤ 4 mm, laser therapy leads to an evident reduction in probing depth but not enough to be statistically significant.

The conclusions of the present study should be considered preliminary and interpreted with caution. Further randomized clinical trials should be conducted to obtain solid conclusions.

## Figures and Tables

**Table 1:** Mean values of PI, PPD, and BOP.

Index	Group	Moment T0	Moment T1	Moment T2
PI (%)	G1	41.07	19.46	5.31
	G2	42.85	-	17.85
PPD (mm)	G1 IL	3.28	2.80	2.33
	G1 IC	3.33	2.85	2.61
	G2	3.38	-	3.23
BOP (score)	G1 IL	2.14	0.52	0.33
	G1 IC	2.19	0.66	0.47
	G2	2.23	-	0.80

T0 = initial examination, T1 = three months after, and T2 = six months after.

## Data Availability

The data presented in this study are available on request from the corresponding authors. The data are not publicly available due to personal protection.
